# Increased hair selenium concentration in hyperlipidemic patients

**DOI:** 10.1111/jcmm.12013

**Published:** 2013-02-13

**Authors:** Péter Fülöp, Ildikó Seres, Zoltán Jenei, Imre Juhász, György Paragh

**Affiliations:** Institute of Internal Medicine, University of Debrecen Medical and Health Science CenterDebrecen, Hungary

**Keywords:** selenium, hyperlipidemia, atherosclerosis

## Abstract

Selenium is an essential trace element with potential anti-atherogenic and antioxidant effects. Experimental data suggest that selenium might be beneficial in the prevention of atherosclerosis and its complications, whereas human epidemiological studies have yielded conflicting results. Data on hair selenium status in hyperlipidemic patients are still lacking. Therefore, we analysed selenium concentrations by X-ray fluorescence in the hair of 81 statin-naïve patients with newly diagnosed Fredrickson-type IIa and IIb hyperlipoproteinemia and compared their data with 43 healthy volunteers. We also assessed the frequency of other classical risk factors of atherosclerosis. Hair selenium levels were found to be significantly higher in hyperlipidemic patients compared with volunteers with normal lipid levels. Also, a significantly increased number of traditional atherosclerosis risk factors were observed in hyperlipidemic patients with hair selenium concentrations above the median in contrast to those with below. Our results suggest that high hair selenium status might be associated with adverse blood lipid profile together with an increased number of traditional risk factors in a selenium-deplete population. These findings warrant further investigations to study the impact of selenium supplementation on the incidence of cardiovascular events.

## Introduction

Selenium is an essential trace mineral that plays an important role in several redox, metabolic and inflammatory pathways in mammals. These processes are also involved in the development of atherosclerosis, in which selenium is suggested to be protective functioning as a selenocysteine incorporated into selenoproteins. To date, at least 25 selenoproteins have been identified [1]. Epidemiological studies have previously established the importance of dyslipidemia and oxidative stress in atherosclerosis, in which oxidized low-density lipoprotein (ox-LDL) is the major culprit by initiating and maintaining the progression of the disease [2–4]. Besides hyperlipidemia, other factors such as hypertension, diabetes mellitus, smoking, obesity and positive cardiovascular family history present the traditional risk factors for atherosclerosis resulting in the increased incidence of cardiovascular events in these individuals.

As it is reviewed elsewhere, previous animal-based studies have shown that—amongst other antioxidants—selenium might reduce oxidative stress and serve as a cytoprotective agent in harmful courses characterized by enhanced reactive oxygen species (ROS) production and/or inflammation, all of which are hallmarks of atherosclerosis [5–7]. Of note, human studies investigating associations between selenium status and atherosclerotic complications have revealed conflicting data. Population-based studies have reported inverse correlations between serum selenium levels and cardiovascular mortality [8–10]; however, other investigations have revealed mixed results about the impact of selenium on incident coronary heart disease [11]. Also, recent studies have found positive correlation between high levels of serum selenium and adverse blood lipid profile or diabetes [12–14]. Furthermore, attention has been drawn to the association of high selenium exposure and increased prevalence of hypertension, together with a more atherogenic lipid profile [15, 16]. It should also be noted that recent data do not support routine administration of selenium in the prevention of cardiovascular disease (CVD) [11].

The majority of the above-mentioned studies have measured serum levels as a marker of selenium status, which one can interpret as a ‘snapshot’ of potential antioxidant capacity provided by this trace element. Other techniques, such as toenail or hair selenium levels might provide an assessment reflecting a longer interval of mineral status in the human body. However, literature employing these techniques specifically aimed to investigate correlations between selenium status and atherosclerotic complications or lipid profile is scarce: only a few studies have been performed on toenail selenium status [17–21] and investigations on hair selenium is even less [22, 23].

To our knowledge, especially in our Central-Eastern European region, there is no literature published about the hair selenium status of hyperlipidemic patients. Also, data are lacking regarding the associations between hair selenium status and the prevalence of traditional risk factors of atherosclerosis. Therefore, we aimed to find out whether hair selenium levels of hyperlipidemic patients would differ from that of healthy individuals and whether hair selenium status would show a correlation with the lipid profile or with the presence of classical risk factors of atherosclerosis in hyperlipidemic patients.

## Materials and Methods

### Study population

We enrolled 81 consecutive patients with newly diagnosed Fredrickson's type IIa or IIb hyperlipoproteinemia that were referred to our lipid clinic at the 1st Department of Internal Medicine, University of Debrecen Medical and Health Science Center, Hungary; and compared their data with 43 healthy volunteers recruited from our department. All participants provided their written informed consent. Study design, analyses and data collection were approved by the institutional ethic board. The study was carried out according to the World Medical Association Declaration of Helsinki. Individuals with active liver, kidney and dermatological scalp diseases, having a diagnosis of type 1 diabetes mellitus or secondary hypertension were excluded from the study. Also, we did not enrol individuals with positive history of CVD or taking lipid lowering drugs, vitamin supplements, selenium or iron containing drugs. None of the investigated participants used medicated shampoos, bleaches or dyes.

Participants completed a physical examination and a questionnaire about their medical history. Metric body mass index (BMI) was calculated as the ratio of the weight measurements in kilogrammes to the square of the height measurements in metres. After a 10-min. rest, systolic blood pressure (SBP) and diastolic blood pressure (DBP) were measured three times 2 min. apart in sitting position. Hypertension was defined as averages of SBP ≥ 140 and/or DBP ≥ 90 mmHg or currently receiving antihypertensive treatment. Patients with the diagnosis of type 2 diabetes mellitus and receiving any type of anti-diabetic treatment were classified as type 2 diabetic. Smoking was defined if the patients declared themselves as current smokers or had stopped <15 years ago. Patients were asked about the health status of their close relatives including their parents, grandparents, siblings, children and grandchildren; family history was considered to be positive for cardiovascular events if the participants had reported incidence any of ischaemic heart disease, acute coronary syndrome, stroke or peripheral arterial disease in their relatives.

### Lipid measurements

After a 12-hr fast, blood was drawn from cubital veins and sera were prepared immediately. Reagents were purchased from Roche Diagnostics, Basel, Switzerland. Lipid fractions were measured by automated methods: serum cholesterol and triglyceride levels were measured by enzymatic, colorimetric tests (cholesterol oxidase-*p*-aminophenazone—CHOD-PAP; and glycerol phosphate oxidase-*p*-aminophenazone—GPO-PAP respectively; Modular P-800 Analyzer; Roche/Hitachi, Basel, Switzerland). High-density lipoprotein cholesterol (HDL-C) and low-density lipoprotein cholesterol (LDL-C) were assessed by homogenous, enzymatic, colorimetric assays (HDL-C plus 3rd generation and LDL-C plus 2nd generation respectively). Apolipoprotein A-1 (Apo A-1) and apolipoprotein B (Apo B) examinations were performed by immunoturbidimetric assays (Tina-quant a Apolipoprotein A-1 ver. 2 and Tina-quant a Apolipoprotein B ver. 2 respectively). The tests were performed according to the recommendation of the manufacturer.

### Selenium measurements

Hair samples were collected with stainless steel scissors from three different places at the occipitonuchal region of the participants, 1 cm away from the scalp. Samples were washed with deionized water to remove topical contaminants. The cut ends of the samples were joined to each other and a 50-mg hair sample was cut and fixed on three special, trace element-free, nylon filaments. Selenium levels were investigated by X-ray fluorescence (XRF) analysis with an automated X-ray emission analytical apparatus of energy resolution of 165 eV (Full Width of Half Maximum, FWHM = 165 eV, MnKα) as it is described elsewhere [24–26]. The characteristic radiation of the sample elements was excited by ring-shaped, 20 GBq and 1000 MBq activity sources containing Fe-55 and I-125 radioisotopes. The X-ray spectrum of the samples was analysed by AXIL software, and selenium concentration (parts per million, ppm) was determined by XRF-BIO software [25].

### Statistical analysis

Statistical analysis was performed by the SAS™ (SAS Institute Inc., Cary, NC, USA) for Windows™ 8.2 computer software. As hair selenium and triglyceride levels showed a skewed distribution, we performed a logarithmic transformation on these variables. Comparisons between groups were analysed by unpaired *t*-tests. Wald–Wolfowitz runs test was applied to analyse the association between *log* hair selenium and the presence of risk factors. *P* < 0.05 was considered statistically significant.

## Results

The characteristics of the study population are shown in Table 1. When compared with the control individuals, serum total cholesterol, LDL-C and apoB levels were measured to be significantly higher in the patients (*P* < 0.001). Serum triglyceride and hair selenium levels showed a skewed distribution with the majority of the values falling into the lower range; however, these lipid and trace mineral concentrations appeared to have a high inter-individual variability; therefore we performed a logarithmic transformation on these variables. As expected, *log* triglyceride was significantly higher in hyperlipidemic patients compared with healthy individuals (*P* = 0.006). Interestingly, however, *log* hair selenium was found to be significantly increased in hyperlipidemic patients in contrast to healthy individuals (*P* = 0.0013).

**Table 1:** Characteristics of the study population

Variables	Controls (*n* = 43)	Patients (*n* = 81)	*P*
Age (years)	52.5 ± 14.4	50.5 ± 11.2	n.s.
Gender (male:female)	20:23	37:44	
BMI (kg/m^2^)	27.7 ± 4.7	27.2 ± 3.5	n.s.
TC (mmol/l)	5.08 ± 0.65	7.42 ± 1.49	<0.001
HDL-C (mmol/l)	1.55 ± 0.65	1.32 ± 0.32	n.s.
LDL-C (mmol/l)	3.03 ± 0.37	5.12 ± 1.25	<0.001
Apo A-1 (g/l)	1.59 ± 0.42	1.53 ± 0.27	n.s.
Apo B (g/l)	0.92 ± 0.21	1.48 ± 0.46	<0.001
Triglyceride (mmol/l)	1.29 (0.99–1.97)	1.92 (1.47–3.05)	
Hair selenium (ppm)	0.4 (0.08–0.94)	1.03 (0.54–2.06)	
*Log* triglyceride	0.28 ± 0.59	0.81 ± 0.64	0.006
*Log* hair selenium	−1.369 ± 2.066	−0.077 ± 1.144	0.0013

Data are expressed as mean ± SD, excluding triglyceride and hair selenium.

Triglyceride and hair selenium are expressed as median and interquartile range.

*P*: control individuals *versus* hyperlipidemic patients.

TC: total cholesterol.

For further analysis, we divided the hyperlipidemic patients into two groups on the basis of their hair selenium status: hyperlipidemic individuals with below and above the median of *log* hair selenium respectively (Table 2). Age, BMI and lipid profile were not significantly different between the two patient groups.

**Table 2:** Characteristics of hyperlipidemic patients with below and above the median of *log* hair selenium

Variables	Below median (*n* = 40)	Above median (*n* = 41)	*P*
Age (years)	50.9 ± 11.7	50.1 ± 10.9	n.s.
Gender (male:female)	17:23	20:21	
BMI (kg/m^2^)	27.0 ± 3.7	27.4 ± 3.3	n.s.
TC (mmol/l)	7.38 ± 1.53	7.45 ± 1.48	n.s.
HDL-C (mmol/l)	1.29 ± 0.33	1.36 ± 0.32	n.s.
LDL-C (mmol/l)	5.17 ± 1.30	5.08 ± 1.22	n.s.
Apo A-1 (g/l)	1.56 ± 0.32	1.49 ± 0.21	n.s.
Apo B (g/l)	1.52 ± 0.52	1.43 ± 0.38	n.s.
*Log* triglyceride	0.79 ± 0.63	0.84 ± 0.66	n.s.

Data are expressed as mean ± SD.

*P*: patients below the median of *log* hair selenium *versus* patients above the median of *log* hair selenium.

TC: Total cholesterol.

We also assessed the prevalence of other traditional risk factors of atherosclerosis across these groups (Table 3). Although none of the differences have reached statistical significance, smoking was tended to be more frequent in patients with high selenium levels: 32% of the hyperlipidemic patients were smokers in the high-selenium group, whereas this ratio appeared to be 20% amongst those who had hair selenium below the median. It should be noted, that hair selenium concentration did not show a significant difference between smoking and non-smoking patients (data not shown). We did not find a similar tendency in the frequencies of other classical risk factors, such as diabetes mellitus, hypertension or positive familial history.

**Table 3:** Frequencies of risk factors in hyperlipidemic patients with below and above the median of *log* hair selenium

Risk factor	Below median (%)	Above median (%)	*P*
Smoking	20	32	n.s
Diabetes mellitus	8	7	n.s.
Hypertension	35	34	n.s.
Positive familial cardiovascular history	43	46	n.s.

Percentage is expressed as proportion of patients carrying the observed risk factors in the corresponding group.

**Fig. 1:**
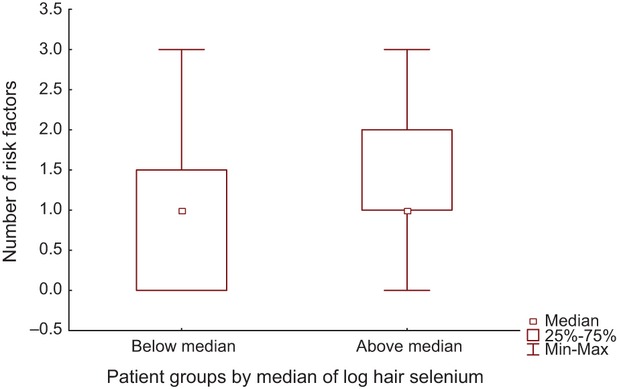
Number of risk factors in hyperlipidemic patients with below and above the median of *log* hair selenium (*P* = 0.0025).

Assessing the prevalence of all risk factors, we found that hyperlipidemic patients in the high-selenium group had significantly more traditional risk factors of atherosclerosis (*P* = 0.0025) compared with those with low hair selenium concentration (Fig. 1). Our results indicate that 50% of the patients with low hair selenium levels possessed one or more risk factors other than hyperlipidemia, whereas this ratio was found to be 75% in individuals with hair selenium levels exceeding the median.

## Discussion

In this study, we determined hair selenium levels and prevalence of some major risk factors of atherosclerosis in newly diagnosed hyperlipidemic patients. We found increased levels of hair selenium in hyperlipidemic patients compared with those with normal lipid levels. Also, our data indicate that hyperlipidemic patients with hair selenium levels above the median carry significantly more traditional risk factors of atherosclerosis in contrast to those hyperlipidemics with hair selenium levels below the median.

Selenium is an essential trace element that is present in soil and enters the food chain through plants. Soil selenium concentration is considered to be low in Europe [27] and high in the United States [28]. Selenium is also known to have a narrow therapeutic window with a large inter-individual variability [29]. Selenium is effective mainly when incorporated into selenoproteins that are involved in several mechanisms such as defence against ROS, inflammation and maybe, cancer [1, 5]. Oxidative stress and inflammation are of major importance in atherosclerosis because they stimulate the formation of ox-LDL resulting in cholesterol accumulation and foam cell formation in the vessel wall [2–4, 6, 7]. Selenium supplementation has been proven to be protective against oxidative injury in human and rodent endothelial cells, too [30, 31]. Based on the potential of selenium and selenoproteins to protect against ROS-induced injury, significant expectations have been raised for the prevention of CVD, however, data are conflicting.

Despite the promising results of basic research, human studies have revealed mixed data regarding the association between selenium exposure and cardiovascular risk. Recent reports have shown that high selenium exposure—especially in selenium-replete populations—has been associated with adverse cardio-metabolic outcome, such as lipid metabolic disorders, together with a higher prevalence of type 2 diabetes mellitus and hypertension. Selenium supplementation has failed to reduce the risk of CVD in previous trials; and selenium administration is not advised recently in the prevention of cardiovascular diseases [13–16]. Although these studies have investigated serum selenium levels and we examined hair selenium concentrations in patients without manifest CVD, our findings corroborate recent data indicating that high selenium exposure might be associated with adverse lipid profile [32]. It should also be considered that we measured total lipid levels; however, composition of lipoprotein particles might be modified during the progression of atherosclerosis [33]; therefore, our results cannot be applied automatically to patients with manifest CVD. Alterations in lipoprotein subpopulations—*i.e*. increased pro-inflammatory HDL levels and/or increased oxidized LDL and small-dense LDL fractions—might modify the outcome of the atherosclerotic process.

The major form of dietary selenium is selenomethionine that cannot be synthesized by humans. Selenocysteine is responsible for the biological activity of selenium and it is also well absorbed. This is also the case for the inorganic forms of the mineral, selenate and selenite that are widely used as dietary supplements. Plant foods, cereals, nuts, meat and seafood are good sources of selenium. Generally, our study population—both healthy controls and hyperlipidemic patients—consumed the same diet containing approximately 30% fat and did not change their dietary habits before serum and hair sampling. It is presumable that not only the dietary factors are responsible for the differences found in our study. In addition, increased selenium intake results in a nonspecific incorporation of selenomethionine into albumin and other proteins [34]; however, the role and metabolic activity of this extra selenium pool are not known yet.

Recent evidence on the impact of selenium on human lipid metabolism is poor. Previous studies have demonstrated that small amounts of selenium are bound to lipoproteins, especially to LDL and very low-density lipoprotein (VLDL) [35, 36]. Hypercholesterolemia might affect the synthesis of selenocysteine, which is an essential compound of selenoproteins, as isopentenylation of selenocysteine tRNA and one step in the formation of cholesterol require the same substrate, isopentenyl pyrophosphate [37]. Also, one might speculate that altered hepatic expression and secretion of selenoprotein P—which contains approximately 60% of total selenium in plasma and transports it to other tissues [5]—might contribute to the increased transport of selenium to the hair in hyperlipidemic patients. Indeed, serum selenoprotein P levels were recently found to be elevated in patients with impaired glucose metabolism [38]. However, it is still unclear, whether the same phenomenon exists in hyperlipidemic patients. Selenium is also known to modify the activity of lipoprotein lipase in rats [39]. Altered tissue-specific activation of the enzyme might also result in elevated hair selenium levels generating an extra selenium pool.

Considering the monthly hair growth rate, hair samples being cut 1 cm away from the scalp represent the mineral status of the last 3 weeks, approximately, which can be a reliable biomarker of selenium status. Based on the median of *log* hair selenium levels, further assessment of the hyperlipidemic patients did not mirror significant differences in the lipid profile of individuals below or above the median of the trace mineral, indicating that lipid measurements might be affected by factors other than selenium exposure in these patients.

Serum selenium level has been reported to be decreased in smoking individuals [40], whereas other studies have shown no relationship between smoking and selenium status [41]. In contrast to these findings, we observed a tendency of smoking being more frequent in patients with high hair selenium levels; however, the difference did not reach statistical significance.

We also found a significantly increased number of traditional atherosclerosis risk factors in hyperlipidemic patients with hair selenium concentrations above the median. Patients were slightly overweight and hyperlipidemic irrespectively of their hair selenium concentrations, which indicate the presence of other factors in the background of this result. Glutathione peroxidase (GPx) isoenzymes are known to be differently reactive to changes in selenium status [5] and erythrocyte GPx activity has been reported to be increased in patients with acute myocardial infarction compared with population controls [17]; therefore, higher hair selenium concentrations and the increased number of risk factors might reflect an activated and a continuously working antioxidant defence in hyperlipidemic patients. Selenium is known to be abundant in food that is also rich in cholesterol, therefore, other lipid metabolic alterations—being either pro- or anti-inflammatory, which are not measured routinely—might lead to an increased hair selenium level. Taking into consideration that we conducted a cross-sectional study on a limited number of individuals and selenium showed a high inter-individual variability, we cannot answer if selenium supplementation would be beneficial for hyperlipidemic patients to avoid subsequent cardiovascular complications. Indeed, current evidence does not support selenium supplementation for the prevention of cardiovascular diseases.

Our findings provide new data regarding hair selenium status in hyperlipidemic patients with different risk factors in a selenium-deplete population and reassure previous results that high selenium levels—together with a higher frequency of traditional risk factors—might contribute to a development of adverse lipid profile and progression of atherosclerosis. Further information on hair selenium status in a prospective study design is of major importance to assess the risk of different cardiovascular outcome in hyperlipidemic patients with various selenium levels.

## References

[1] Kryukov GV, Castellano S, Novoselov SV (2003). Characterization of mammalian selenoproteomes. Science.

[2] Munro JM, Cotran RS (1988). The pathogenesis of atherosclerosis: atherogenesis and inflammation. Lab Invest.

[3] Luc G, Fruchart JC (1991). Oxidation of lipoproteins and atherosclerosis. Am J Clin Nutr.

[4] Ross R (1993). The pathogenesis of atherosclerosis: a perspective for the 1990s. Nature.

[5] Reeves MA, Hoffmann PR (2009). The human selenoproteome: recent insights into functions and regulation. Cell Mol Life Sci.

[6] Hulsmans M, Holvoet P (2010). The vicious circle between oxidative stress and inflammation in atherosclerosis. J Cell Mol Med.

[7] Lü JM, Lin PH, Yao Q (2010). Chemical and molecular mechanisms of antioxidants: experimental approaches and model systems. J Cell Mol Med.

[8] Salonen JT, Alfthan G, Huttunen JK (1982). Association between cardiovascular death and myocardial infarction and serum selenium in a matched-pair longitudinal study. Lancet.

[9] Lubos E, Sinning CR, Schnabel RB (2010). Serum selenium and prognosis in cardiovascular disease: results from the AtheroGene study. Atherosclerosis.

[10] Eaton CB, Abdul Baki AR, Waring ME (2010). The association of low selenium and renal insufficiency with coronary heart disease and all-cause mortality: NHANES III follow-up study. Atherosclerosis.

[11] Flores-Mateo G, Navas-Acien A, Pastor-Barriuso R (2006). Selenium and coronary heart disease: a meta-analysis. Am J Clin Nutr.

[12] Stranges S, Laclaustra M, Ji C (2010). Higher selenium status is associated with adverse blood lipid profile in British adults. J Nutr.

[13] Laclaustra M, Stranges S, Navas-Acien A (2010). Serum selenium and serum lipids in US adults: National Health and Nutrition Examination Survey (NHANES) 2003–2004. Atherosclerosis.

[14] Stranges S, Galletti F, Farinaro E (2011). Associations of selenium status with cardiometabolic risk factors: an 8-year follow-up analysis of the Olivetti Heart Study. Atherosclerosis.

[15] Laclaustra M, Navas-Acien A, Stranges S (2009). Serum selenium concentrations and hypertension in the US Population. Circ Cardiovasc Qual Outcomes.

[16] Bleys J, Navas-Acien A, Stranges S (2008). Serum selenium and serum lipids in US adults. Am J Clin Nutr.

[17] Kok FJ, Hofman A, Witteman JC (1989). Decreased selenium levels in acute myocardial infarction. JAMA.

[18] Kardinaal AF, Kok FJ, Kohlmeier L (1997). Association between toenail selenium and risk of acute myocardial infarction in European men. The EURAMIC Study. European antioxidant myocardial infarction and breast cancer. Am J Epidemiol.

[19] Yoshizawa K, Ascherio A, Morris JS (2003). Prospective study of selenium levels in toenails and risk of coronary heart disease in men. Am J Epidemiol.

[20] Xun P, Liu K, Morris JS (2010). Longitudinal association between toenail selenium levels and measures of subclinical atherosclerosis: the CARDIA trace element study. Atherosclerosis.

[21] Xun P, Liu K, Morris JS (2010). Associations of toenail selenium levels with inflammatory biomarkers of fibrinogen, high-sensitivity c-reactive protein, and interleukin-6: the CARDIA Trace Element Study. Am J Epidemiol.

[22] Thimaya S, Ganapathy SN (1982). Selenium in human hair in relation to age, diet, pathological condition and serum levels. Sci Total Environ.

[23] Djujić IS, Jozanov-Stankov ON, Milovac M (2000). Bioavailability and possible benefits of wheat intake naturally enriched with selenium and its products. Biol Trace Elem Res.

[24] Bacsó J, Uzonyi I (1988). Development of an automatic sample changer for XRF measurements. Izotopenpraxis - Isotopes Environ Health Stud.

[25] Uzonyi I (1988). A new XRF method for the analysis of biological samples. Izotopenpraxis - Isotopes Environ Health Stud.

[26] Shenberg C, Mantel M, Izak-Biran T (1988). Rapid and simple determination of selenium and other trace elements in very small blood samples by XRF. Biol Trace Elem Res.

[27] Brown KM, Arthur JR (2001). Selenium, selenoproteins and human health: a review. Public Health Nutr.

[28] Bleys J, Navas-Acien A, Guallar E (2007). Serum selenium and diabetes in US adults. Diabetes Care.

[29] Whanger P, Vendeland S, Park YC (1996). Metabolism of subtoxic levels of selenium in animals and humans. Ann Clin Lab Sci.

[30] Thomas JP, Geiger PG, Girotti AW (1993). Lethal damage to endothelial cells by oxidized low density lipoprotein: role of selenoperoxidases in cytoprotection against lipid hydroperoxide- and iron-mediated reactions. J Lipid Res.

[31] Huang K, Liu H, Chen Z (2002). Role of selenium in cytoprotection against cholesterol oxide-induced vascular damage in rats. Atherosclerosis.

[32] Stranges S, Tabák AG, Guallar E (2011). Selenium status and blood lipids: the cardiovascular risk in Young Finns study. J Intern Med.

[33] Schultz JR, Verstuyft JG, Gong EL (1993). Protein composition determines the anti-atherogenic properties of HDL in transgenic mice. Nature.

[34] Daniels LA (1996). Selenium metabolism and bioavailability. Biol Trace Elem Res.

[35] Ducros V, Laporte F, Belin N (2000). Selenium determination in human plasma lipoprotein fractions by mass spectrometry analysis. J Inorg Biochem.

[36] Burk RF (1974). Invivo Se-75 binding to human plasma-proteins after administration of Se-75 O3 2-. Biochim Biophys Acta.

[37] Moosmann B, Behl C (2004). Selenoprotein synthesis and side-effects of statins. Lancet.

[38] Yang SJ, Hwang SY, Choi HY (2011). Serum selenoprotein P levels in patients with type 2 diabetes and prediabetes: implications for insulin resistance, inflammation, and atherosclerosis. J Clin Endocrinol Metab.

[39] Ueki H, Ohkura Y, Motoyashiki T (1993). Increase in lipoprotein lipase activity in isolated rat adipose tissue by selenate. Biol Pharm Bull.

[40] Wei W, Kim Y, Boudreau N (2001). Association of smoking with serum and dietary levels of antioxidants in adults: NHANES III, 1988–1994. Am J Public Health.

[41] Robinson MF, Campbell DR, Sutherland WH (1983). Selenium and risk factors for cardiovascular disease in New Zealand. N Z Med J.

